# Accelerated Evolution of Schistosome Genes Coding for Proteins Located at the Host–Parasite Interface

**DOI:** 10.1093/gbe/evu287

**Published:** 2015-01-06

**Authors:** Gisele S. Philippsen, R. Alan Wilson, Ricardo DeMarco

**Affiliations:** ^1^Departamento de Física e Ciência Interdisciplinar, Instituto de Física de São Carlos, Universidade de São Paulo, São Carlos, São Paulo, Brazil; ^2^Centre for Immunology and Infection, Department of Biology, University of York, United Kingdom

**Keywords:** nonsynonymous mutations, gene evolution, gene duplication, vaccine candidates

## Abstract

Study of proteins located at the host–parasite interface in schistosomes might provide clues about the mechanisms utilized by the parasite to escape the host immune system attack. Micro-exon gene (MEG) protein products and venom allergen-like (VAL) proteins have been shown to be present in schistosome secretions or associated with glands, which led to the hypothesis that they are important components in the molecular interaction of the parasite with the host. Phylogenetic and structural analysis of genes and their transcripts in these two classes shows that recent species-specific expansion of gene number for these families occurred separately in three different species of schistosomes. Enrichment of transposable elements in MEG and VAL genes in *Schistosoma mansoni* provides a credible mechanism for preferential expansion of gene numbers for these families. Analysis of the ratio between synonymous and nonsynonymous substitution rates (d*N*/d*S*) in the comparison between schistosome orthologs for the two classes of genes reveals significantly higher values when compared with a set of a control genes coding for secreted proteins, and for proteins previously localized in the tegument. Additional analyses of paralog genes indicate that exposure of the protein to the definitive host immune system is a determining factor leading to the higher than usual d*N*/d*S* values in those genes. The observation that two genes encoding *S. mansoni* vaccine candidate proteins, known to be exposed at the parasite surface, also display similar evolutionary dynamics suggests a broad response of the parasite to evolutionary pressure imposed by the definitive host immune system.

## Introduction

Schistosomes are blood flukes that are the causative agents of schistosomiasis. Some species of this genus establish a chronic infection in the human host that can persist for decades, despite continuing exposure of the parasite to the host immune attack. Definition of the molecular mechanisms underlying the ability of the parasite to escape the host immune system is of crucial importance. Description of proteins located at the tegument surface, the outermost layer of the mature worm, or secreted by the parasite (Knudsen et al. 2005; Braschi and Wilson 2006; Curwen et al. 2006; Mathieson and Wilson 2010; Hall et al. 2011; Li et al. 2013) represented a first step toward characterization of relevant molecular systems involved in this process.

Study of egg and larval schistosomula secretions from *Schistosoma mansoni* allowed the detection of proteins derived from two different families of micro-exon genes (MEGs) (DeMarco et al. 2010). MEG protein products and transcripts were also found to be associated with glands and epithelia exposed to the external environment in stages infecting the definitive host (DeMarco et al. 2010; Li et al. 2013). MEGs display an unusual structure in which most of the coding region of the gene is represented by very small symmetrical exons. This allows for the use of alternative splicing as a mechanism to generate a pool of very similar proteins differing by a few amino acids (DeMarco et al. 2010). MEGs have been classified into 25 different families based on their protein sequence (DeMarco et al. 2010; Almeida et al. 2012) and appear to be restricted to platyhelminths. All MEG products display a signal peptide and are predicted to be secreted or anchored in the plasma membrane of the parasite. Moreover, the transcription levels of several MEGs are strongly upregulated during the definitive host invasion process (Parker-Manuel et al. 2011). The presence of a very specialized mechanism for generation of a variable pool of related proteins led to the hypothesis that MEGs were tailored during evolution to provide a variable set of antigens that would act as a smokescreen to the host immune system (DeMarco et al. 2010).

Venom allergen-like (VAL) proteins display an Sperm-Coating Protein (SCP) domain which is widely distributed throughout several branches of evolution. In helminths, these proteins are known to be secreted during infection, displaying angiogenic proprieties *(*Tawe et al. 2000), inducing Neutrophil recruitment (Bower et al. 2008) and inhibiting platelet aggregation (Del Valle et al. 2003). Twenty-eight VALs have been described in *S. mansoni,* with several of them showing a transcription profile upregulated during the stages that invade the definitive host (Chalmers et al. 2008). VALs of metazoans can be divided into two different groups: Group 1 VALs display several conserved cysteines, indicative of disulphide bond formation, and signal peptides, whereas Group 2 VALs lack such features. These features suggest that Group 1 VALs are extracellular proteins whereas Group 2 VALs are intracellular ones (Chalmers et al. 2008). Indeed, there are descriptions of proteins with SCP domains from Group 1 from several organisms that were detected in the extracellular space (Hawdon et al. 1999; Olson et al. 2001; Milne et al. 2003). Three VALs from Group 1 have been detected in *S. mansoni* cercarial secretions (Curwen et al. 2006) and another was specifically transcribed in the esophageal gland of adult worms (Rofatto et al. 2012).

Comparison of two evolutionarily related sequences allows the measurement of the ratio between nonsynonymous/synonymous substitution rates (d*N*/d*S*), which would present a value of 1 in a scenario of neutral changes. Divergences from this value would reflect the effect of evolutionary pressures, those above or below the threshold representing a process of positive or negative selection of amino acid substitutions in a protein, respectively. Several genes coding for proteins involved in evading the host immune system have been revealed as subject to positive selection by the evaluation of the d*N*/d*S* ratio in their coding regions (Yang and Bielawski 2000). Among them are several polymorphic surface proteins of another blood dwelling parasite, *Plamodium falciparum,* which provide evidence of an evolutionary pressure from host immune defenses on those proteins (Hughes and Hughes 1995). To date, no study has been performed in schistosomes to verify whether a similar process occurred in proteins involved in the host–parasite interaction.

## Materials and Methods

### Analysis of MEG Copies from *S. mansoni* in *Schistosoma japonicum* and *Schistosoma haematobium*

MEG and VAL protein sequences from *S. mansoni* were used as queries for a tBLASTn search for ortholog transcript sequences against a database comprising available transcript sequences (including publicly available RNAseq assembly and expressed sequence tag [EST] data) as well as gene predictions derived from the genomes of *S. haematobium* (Young et al. 2012) and *S. japonicum* (*Schistosoma japonicum* Genome Sequencing and Functional Analysis Consortium 2009). Alignments with *e* value lower than e-10 were considered positive hits, representing possible MEG orthologs in those species. MEG sequences for each family were manually inspected and compared by BLASTn to remove redundant sequences. For MEG families without transcript evidence in *S. haematobium* or *S. japonicum*, a BLASTn search of S*. mansoni* transcript sequences against the *S. haematobium* or *S. japonicum* genome sequences was performed and alignments with an *e* value lower than e-5 were interpreted as the presence of an orthologous exon.

Sequences of full-length proteins were used for phylogenetic analysis in the MEG families where transcript sequences for all detected members were available. In the cases where transcript data were unavailable, nucleotide sequences from one long conserved exon were used. VAL phylogenetic analysis was performed using the whole SCP (SMART00198) domain derived from transcript sequences, disregarding partial domains. Analysis was performed using the Bayesian inference method implemented in MrBayes (v3.1.2). All analyses were run using default parameters, except for the command “prset aamodelpr=mixed” in analysis utilizing protein sequences. Analyses were stopped after 1 million generations, with samplings every 100th generation. Tree information was summarized, discarding the first 250,000 generations, utilizing the “sumt burnin=2500” command. Resulting trees were visualized using the TreeView program (Page 1996). Analysis of phylogenetic trees allowed the detection of monophyletic groups with sequences from a single species and high posterior probability at the base of the branch containing this group, which was counted as resulting from a recent duplication event.

### Analysis of the Enrichment of Transposable Elements in MEG and VAL Gene Family Members

To annotate transposable element (TE) copies, we performed a BLASTn search (hits cutoff *e* value < 10^−^^10^) of TE sequences against the current version of the *S. mansoni* genome (Protasio et al. 2012). Hits positioned on the same chromosome, belonging to the same TE family, in the same orientation, with a distance lower than 100 bp between them and having colinearity with respect to a TE sequence were considered as a single insertion. This approach was adopted to avoid an incorrect overestimation in the number of mobile element copies. Finally, overlapping insertions larger than 50 bp were removed by selecting the copy with higher score.

VAL genes and MEGs with genomic coordinates defined for the whole coding region plus their environs (region up to 1 kb upstream from the annotated gene start and up to 1 kb downstream from the annotated gene end) were considered for analysis of enrichment. For each TE family, an observed frequency in the considered regions of MEGs or VAL genes was compared with the expected frequency. The expected copy number was estimated by the binomial model with parameters *n* and *p* (*T* ∼ *b*(*n*,*p*)), where *n* is the copy number in the entire genome for the TE family and *p* is the probability of the insertion being located in the genomic regions under study. It was assumed that each genomic position has the same probability of containing a TE insertion. The binomial test (normal approximation) was applied to examine the statistical significance of the observed TE copy number in the expected TE copy number probability distribution (*P* value < 0.05).

The TE families that showed an observed frequency higher than expected with statistical significance in the first analysis were subjected to a second analysis involving an empirical simulation. In each round, we generated a number of random genomic regions, equivalent in number and extension to the considered genomic regions of the MEGs or VAL genes. In total, 30,000 simulation rounds were performed to obtain a distribution of copy numbers of the corresponding TE family in the sampled genomic regions. This approach allowed the comparison between the observed copy number of the TE family in the considered genomic regions of the MEGs or VAL genes against equivalent random portions into the genome.

### Analysis of d*N*/d*S* in VAL Genes, MEGs, Vomitus Proteins, and Vaccine Candidates

VALs and MEGs with multiple copies in *S. mansoni* had their orthologs determined based on analysis of syntenic blocks of their genomes based on comparisons of *S. mansoni* genome against *S. haematobium* and S*. japonicum* genomes using SatsumaSynteny program (Grabherr et al. 2010). Determination of syntenic VAL genes pairs of *Caenorhabditis elegans* and *Caenorhabditis briggsae* was obtained at EnsemblMetazoa site (http://metazoa.ensembl.org/index.html, last accessed January 8, 2015). Single copy genes in *S. mansoni* had their ortholog chosen based on bidirectional best hits. Pairs of control gene orthologs were provided based on bidirectional best hits in BLASTp searches utilizing the predicted proteins from the three organisms derived from genome sequencing. In the case of the control sample, only genes that form a perfect triangulation of bidirectional best hits between the three species were selected. This resulted in a set of 5,710 predicted transcript sequences from each species. Control genes encoding secreted proteins were selected from the control subset based on SignalP analysis on *S. mansoni*-predicted proteins. A set of transcript sequences for proteins exposed on the *S. mansoni* parasite surface was retrieved, based on information provided in Braschi and Wilson (2006); the best hit from a BLASTp search in databases of transcripts from *S. haematobium* and *S. japonicum* was then selected as the ortholog protein in each species.

A database of nonredundant MEG and VAL transcripts and their derived proteins was separately produced for *S. mansoni, S. japonicum*,** and *S. haematobium*. In the case of VALs, due to the large number of hits obtained, a nonredundant set was obtained by assembly using fasta2phd script and phredPhrap to remove redundancy. Pairs of paralogs were defined by a BLASTp search of each MEG and VAL against a nonredundant MEG and VAL protein database from the organism in which it originated. Alignments with *e* value lower than e-10 were used to assign pairs of paralogs. These were considered as resulting from recent gene duplications when the proteins forming the pairs were part of the same monophyletic group containing only sequences from the same organism in the phylogeny analysis. A list of the paralogous sequences considered is provided in supplementary table S1, Supplementary Material online. The two families of recently expanded genes in schistosomes coding for cytoplasmic proteins, Tubulin epsilon chain and Dynein light chain (Silva et al. 2012), had their *S. mansoni* paralogs compared and resulting values were used as benchmark.

Sequences from three conserved exons of several schistosome species were obtained from preliminary genome sequences made available by the parasite genomic group at the Welcome Trust Sanger institute (ftp://ftp.sanger.ac.uk/pub/pathogens/HGI/, last accessed January 8, 2015). Alignment of Sm29 protein sequences from *S. mansoni* or *S. haematobium* against the preliminary genome sequence was performed using tBLASTn program allowed definition of a portion of the genome containing exons of Sm29 orthologs in these genomes. Exons boundaries were refined by manual search of canonical acceptor and donor splicing sites.

Pairs of orthologs or paralogs were aligned using the BLASTp algorithm and only the protein sequences from regions contained in this alignment were considered homologous and used for global alignment with muscle v 3.8.31 (Edgar 2004). The resulting alignment was then converted to an equivalent nucleotide alignment using the RevTrans 1.4 standalone tool (Wernersson and Pedersen 2003). d*N*/d*S* values were then calculated based on the resulting nucleotide alignment using Wina 0.36 (Endo et al. 1996), with the total length of the alignment as window size, thus resulting in a single d*S* and d*N* measurement for the entire alignment.

Significance of differences between data sets was calculated using Wilcoxon signed-rank test implemented in R, using the option paired=FALSE.

## Results

### MEGs and VALs Display Multiple Species-Specific Gene Duplications in Schistosomes

Both MEGs and VALs are classes of genes coding for proteins that are exposed to the host immune system and display multiple members. To better understand the evolution of these genes, we performed an analysis to characterize the organization of the two classes of genes in the three species of schistosomes with described genomes: *S. mansoni*, *S. japonicum*,** and *S. haematobium*. We used the protein sequence of the members from the 25 previously described MEG families in *S. mansoni* (DeMarco et al. 2010; Almeida et al. 2012) in a tBLASTn search against a comprehensive transcript database, including complete transcripts, ESTs and RNAseq data, from *S. haematobium* and *S. japonicum.* This allowed the retrieval of orthologs for ten and eight MEG families displaying extensive sequence similarity, from *S. haematobium* and *S. japonicum,* respectively. Mapping of these transcripts back into their respective genomes allowed us to verify the number of copies in each of the genomes (table 1). In addition, a BLASTn search using *S. mansoni* MEGs transcripts for which equivalent transcripts have not been found in the other species as queries and genome assemblies as databases allowed the detection of at least one homologous long flanking exon for members of 13 additional families in *S. haematobium* and 2 in *S. japonicum* (table 1), which is strongly suggestive of the existence of homologous genes for these families in the two species. This indicates that at least 23 of 25 MEG families described in *S. mansoni* have equivalents in the *S. haematobium* genome and that 10 of them are confirmed as transcriptionally active. In *S. japonicum* evidence for the presence of 12 MEG families was found, with nine displaying transcripts. Phylogenetic analysis of the members of each MEG family allowed detection of several cases where a monophyletic group containing only members in the same species was verified (examples shown in supplementary fig. S1, Supplementary Material online), which is suggestive of species-specific gene duplications (table 1).

**Table 1:** Summary of MEG Families Distribution

MEG Family	No. of Members Sma^a^	No. of Members Sha^a^	No. of Members Sja^a^	No. of Species- Specific Duplication Events^b^
Families detected with transcript evidence in *Schistosoma mansoni* and other species				
1	3(C)	1	0	1
2	6(C) + 3(C) + 2(C) + 2(C) + 1	2(C) + 2(C) + 3	2	7
3	4(C)	1	3(C) + 1	5
4	2	2(C) + 1	3(C)	2
5	1	1	1	NA^c^
8	2	2	4	2
9	1	2	4	4
11	1	1	1	NA^c^
14	1	1	2(C) + 2	2
15	1	1	0	NA^c^
17	3(C) + 19	2(C) + 2(C) + 1	0	25
21	1	1	1	NA^c^
24	1	1	0	NA^c^
Families detected in genome, but transcript evidence only in *S. mansoni*				
				
6	3	1	1	2
7	1	1	0	NA^c^
10	2(C)	2(C)	0	2
12	1	2	0	NA^c^
13	1	1	2	1
16	1	1	0	NA^c^
18	5(C)	0	0	NA^c^
19	2(C)	1	0	NA^c^
20	1	0	0	NA^c^
22	1	1	1	NA^c^
23	1	1	0	NA^c^
25	15	3	0	16

Note.—Sma, *Schistosoma mansoni*; Sha, *Schistosoma haematobium*; Sja, *Schistosoma japonicum.*

^a^Number of genes in each organisms represented next to a (C) indicate a cluster of genes in tandem. Genes were considered to form a cluster when the distance between each to the neighbor gene of the same class was no greater than 50 kb.

^b^A duplication was considered species-specific if the node representing it is within a monophyletic group of sequences from the same organism in the phylogenetic analysis.

^c^NA-not applicable.

### Group 1 VAL Genes Tend to Display Fewer and Shorter Introns than Those of Group 2

**Fig. 1.—:**
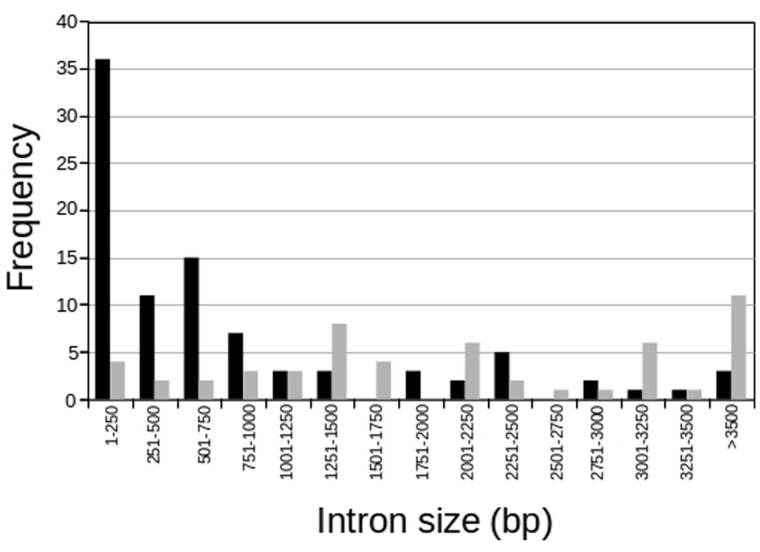
Distribution of intron sizes in VAL genes from Group 1 (black) and Group 2 (gray).

Analysis of VAL gene structure in *S. mansoni* indicates that Group 1 VALs tend to have short introns between coding exons, with a considerable fraction of the introns smaller than 250 bp length (fig. 1). In contrast, Group 2 VALs display a very different size distribution with a higher frequency of much larger introns. Moreover, in all 23 Group 1 VALs, the coding region is represented by five exons, whereas five members of Group 2 VALs have a coding region represented by more exons (7, 8, 9, 10, and 38 exons).

### MEG and VAL Genes in the *S. mansoni* Genome Are Enriched for TE Insertions

An analysis of the frequency of TE was possible for several MEG and VAL genes and their immediate environs only in the *S. mansoni* genome as few *S. japonicum* and *S. haematobium* MEG and VAL gene locus boundaries are completely defined. This is due to genome incompleteness and lack of full transcript sequences, thus preventing a robust analysis. Moreover, a larger collection of TE sequences has been described for *S. mansoni*.

Assuming an equal chance of insertion of TEs throughout the genome, a higher than expected frequency was verified for two Short Interspersed Elements (SINE) elements, in both MEG and VAL genes (supplementary table S2, Supplementary Material online). They are the previously described SmAlpha and Sm elements (Ferbeyre et al. 1998). Both are small, noncoding elements displaying a hammerhead ribozyme structure, similar to that previously described for viroid elements. In addition, the MEG environs also display a significantly higher frequency of Perere-3, a non-long terminal repeat (non-LTR) TE (DeMarco et al. 2005).

**Fig. 2.—:**
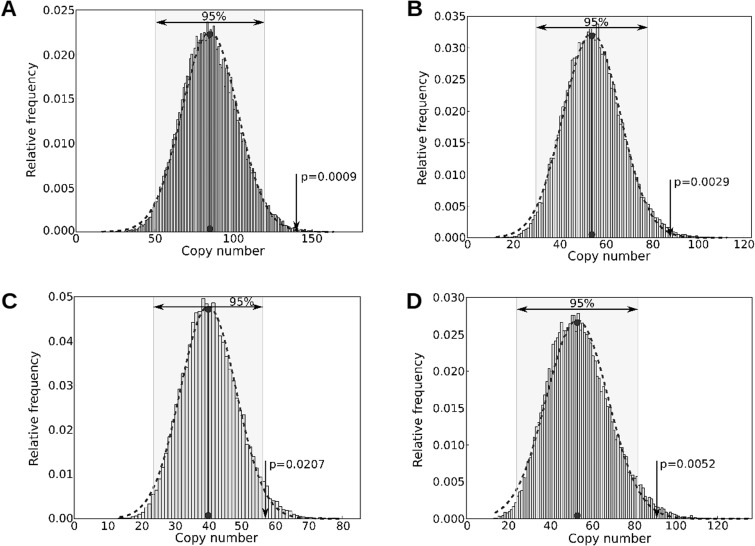
Analysis of enrichment of TEs in VAL and MEG genes and their environs in the *S. mansoni* genome. Histograms represent the distribution of the number of TEs sampled in 30,000 simulations in which random genomic regions, equivalent to genomic regions containing the MEGs or VAL genes in number and extension, were analyzed. Dashed lines indicate a normal distribution curve fitted to the control data histogram and the gray background represents the 95% confidence interval based on the normal distribution. The vertical arrow indicates the observed number of TEs registered in the MEG or VAL genes and its associated *P* value, assuming this normal distribution. (*A*) Analysis of Sm elements in MEGs. (*B*) Analysis of Sm-alpha elements in MEGs. (*C*) Analysis of Perere-3 elements in MEGs. (*D*) Analysis of Sm elements in VALs.

A more sophisticated analysis utilizing empirical simulations was performed for the TE families determined as statistically overrepresented in the preliminary analysis. Genomic portions, having exactly the same profile with regard to size and number as those representing the sampled gene families, were randomly chosen in the *S. mansoni* genome and the number of TE copies computed (fig. 2). Assuming a normal distribution of the frequency of sampled elements from the control regions, the frequency of TE elements in the MEG or VAL genes and their environs is seen to be well above that randomly expected, with a *P* value lower than 0.05 for all cases analyzed (fig. 2), except for SmAlpha enrichment in VAL genes that did not achieve statistical significance (data not shown).

### MEGs and VAL Ortholog Genes Display High d*N*/d*S* Values

**Fig. 3.—:**
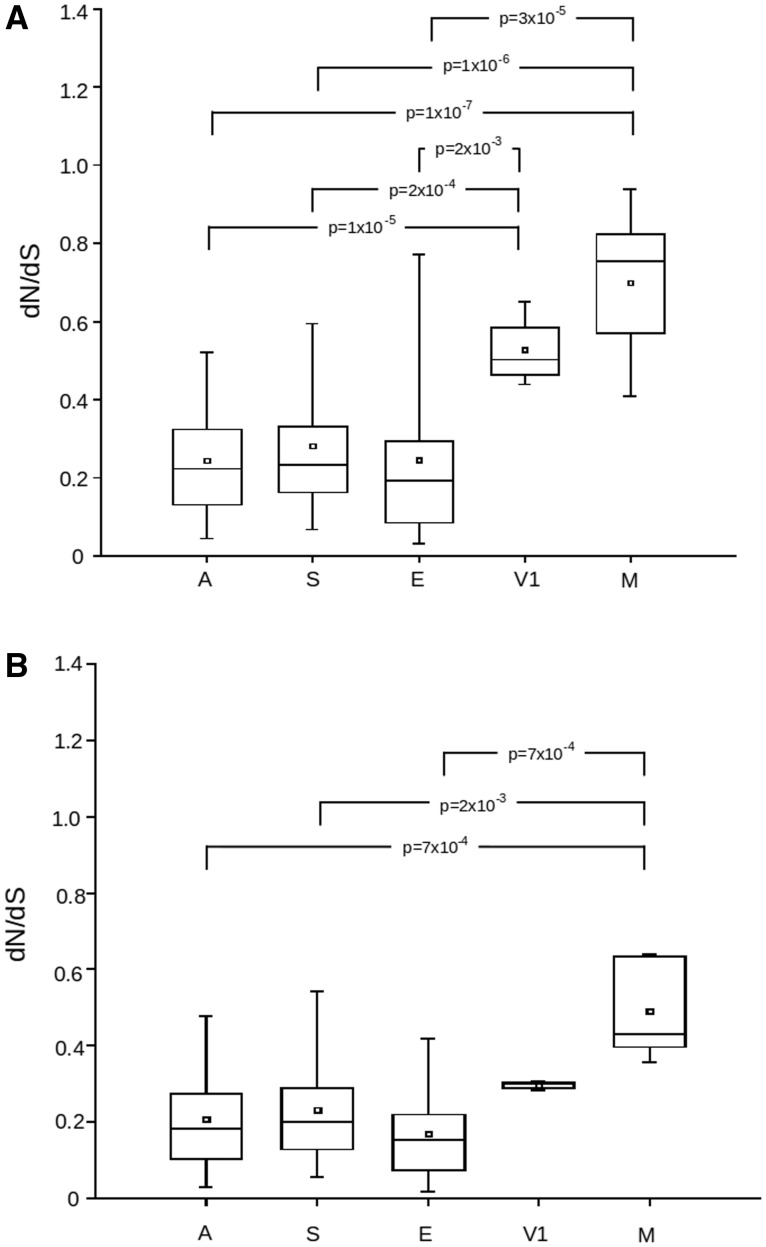
Distribution of d*N*/dS values for different data sets of ortholog genes. Box plots displaying d*N*/d*S* distributions relative to comparisons between pairs of *S. mansoni* × *S. haematobium* (*A*) or *S. mansoni* × *S. japonicum* (*B*) orthologs. A, all ortholog genes (Sm × Sh: *n* = 5,611; Sm × Sj: *n* = 5,654); S, ortholog genes coding for secreted proteins (Sm × Sh: *n* = 247; Sm × Sj: *n* = 248); E, ortholog genes coding for proteins located at *S. mansoni* tegument surface(Sm × Sh: *n* = 21; Sm × Sj: *n* = 21); V1, VAL orthologs from Group 1 (Sm × Sh: *n* = 7; Sm × Sj: *n* = 3); M, MEG orthologs (Sm × Sh: *n* = 11; Sm × Sj: *n* = 5). *P* values calculated using Wilcoxon signed-rank test for different comparisons between data sets are displayed.

In order to verify whether MEG and VAL sequences were subject to any detectable evolutionary pressure, measurements of the ratio between nonsynonymous/synonymous (d*N*/d*S*) substitution rates were performed by comparing *S. mansoni* coding sequences with those of *S. haematobium* and *S. japonicum* orthologs (fig. 3*A* and *B*, respectively). The distribution of d*N*/d*S* values of MEG and VAL gene orthologs was compared with a control distribution obtained for all genes coding for proteins with orthologs in the three species and a subset of the control genes coding for proteins with a detectable signal peptide. In both control data sets, only comparisons between proteins with clear orthologs in all three species were performed. In addition, values of d*N*/d*S* distribution for comparisons involving genes coding for proteins previously shown to be on the outer surface of the *S. mansoni* tegument (Braschi and Wilson 2006) and their orthologs in the other two species were obtained. It is notable that both MEGs and VAL genes display values for d*N*/d*S* that are significantly higher than any of those three control data sets in comparisons between *S. mansoni* and *S. haematobium*, with MEGs displaying noticeably higher values than VAL genes. In comparison between *S. mansoni* and *S. japonicum* orthologs, MEGs still display a significant difference of d*N*/d*S* in relation to the control groups, but differences between VAL genes and control groups are not statistically relevant. Comparisons of values for the control groups in the *S. mansoni* × *S. haematobium* data set are very similar to those observed in *S. mansoni* × *S. japonicum*, indicating that evolutionary pressure on most proteins throughout the evolution of this genus is relatively constant. However, it is notable that d*N*/d*S* values for MEGs and VAL genes are consistently higher in *S. mansoni* × *S. haematobium* comparisons relative to *S. mansoni* × *S. japonicum* comparisons, suggesting the presence of an additional evolutionary factor that created a stronger evolutionary pressure after the divergence of *S. mansoni* and *S. haematobium.*

**Fig. 4.—:**
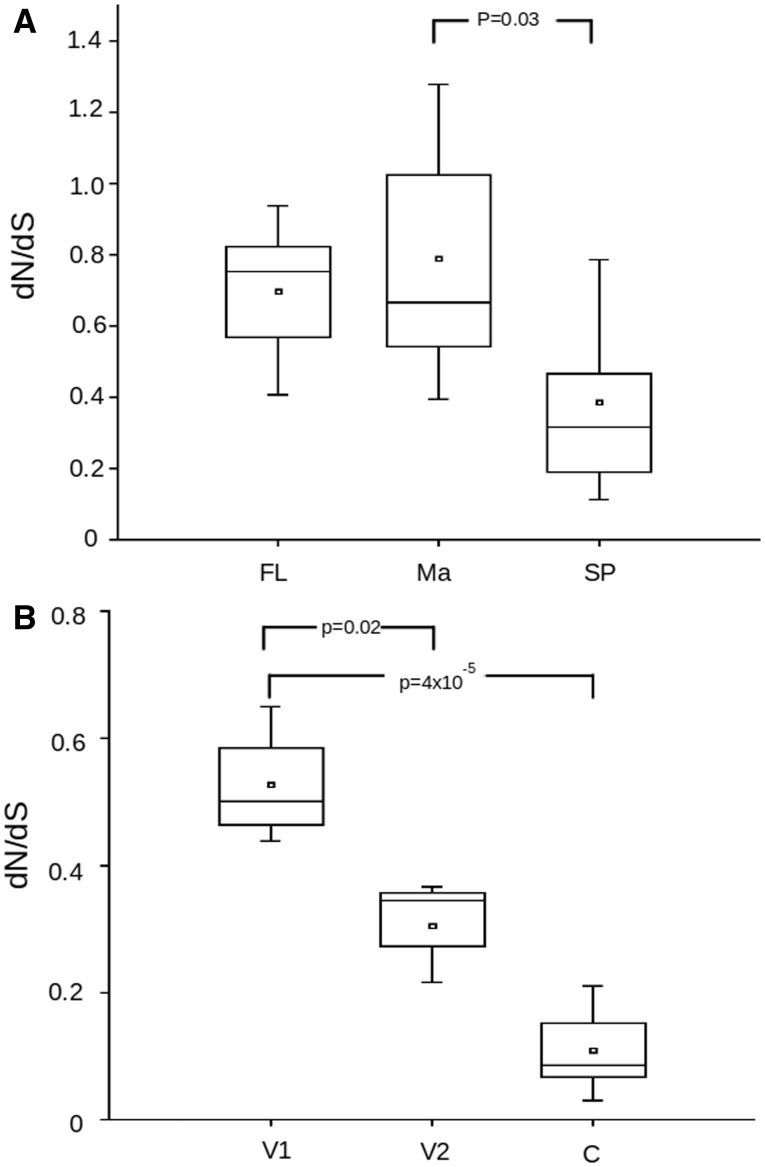
Comparisons of d*N*/d*S* values for data sets corresponding to exposed or internal protein portions. (*A*) Distribution of d*N*/d*S* values between pairs of *S. mansoni* × *S. haematobium* MEG orthologs considering: FL, full-length conserved sequence (*n* = 11); Ma, the portion of the gene corresponding to the conserved mature peptide (*n* = 11); SP, the portion of the gene corresponding to the conserved signal peptide (*n* = 5). (*B*) Distribution of d*N*/d*S* values between pairs of *S. mansoni* × *S. haematobium* VALs orthologs from Group 1 (V1; *n* = 7) and Group 2 (V2; *n* = 3) and from comparisons of *C. elegans* × *C. briggsae VALs* genes coding for proteins with signal peptide (C; *n* = 12). *P* values calculated using Wilcoxon signed-rank test for different comparisons between data sets are displayed.

A more discriminating analysis of comparisons between *S. mansoni* and *S. haematobium* genes was performed by separating the portions of the genes corresponding to the mature MEG protein and its signal peptide portion. This allowed us to establish that these two regions display different evolutionary dynamics (fig. 4*A*), with the gene portion coding for the mature protein displaying significantly higher d*N*/d*S* values than the signal peptide portion. The mature region of *MEG-1*, *MEG-9*, and *MEG-15* displayed values of d*N*/d*S* of greater than 1 (1.14, 1.10, and 1.11, respectively), thus it is possible to consider that they are under positive selection.

Analysis of *S. mansoni* and *S. haematobium* VAL genes from groups 1 and 2 was performed and resulted in a significant difference of d*N*/d*S* between those two groups, with Group 1 genes exhibiting much higher values (fig. 4*B*).

### *Schistosoma mansoni* VAL Paralogs Gene Pairs Coding for Proteins Exposed to the Definitive Host Display Higher d*N*/d*S* than Those Exposed to the Intermediate Host

**Fig. 5.—:**
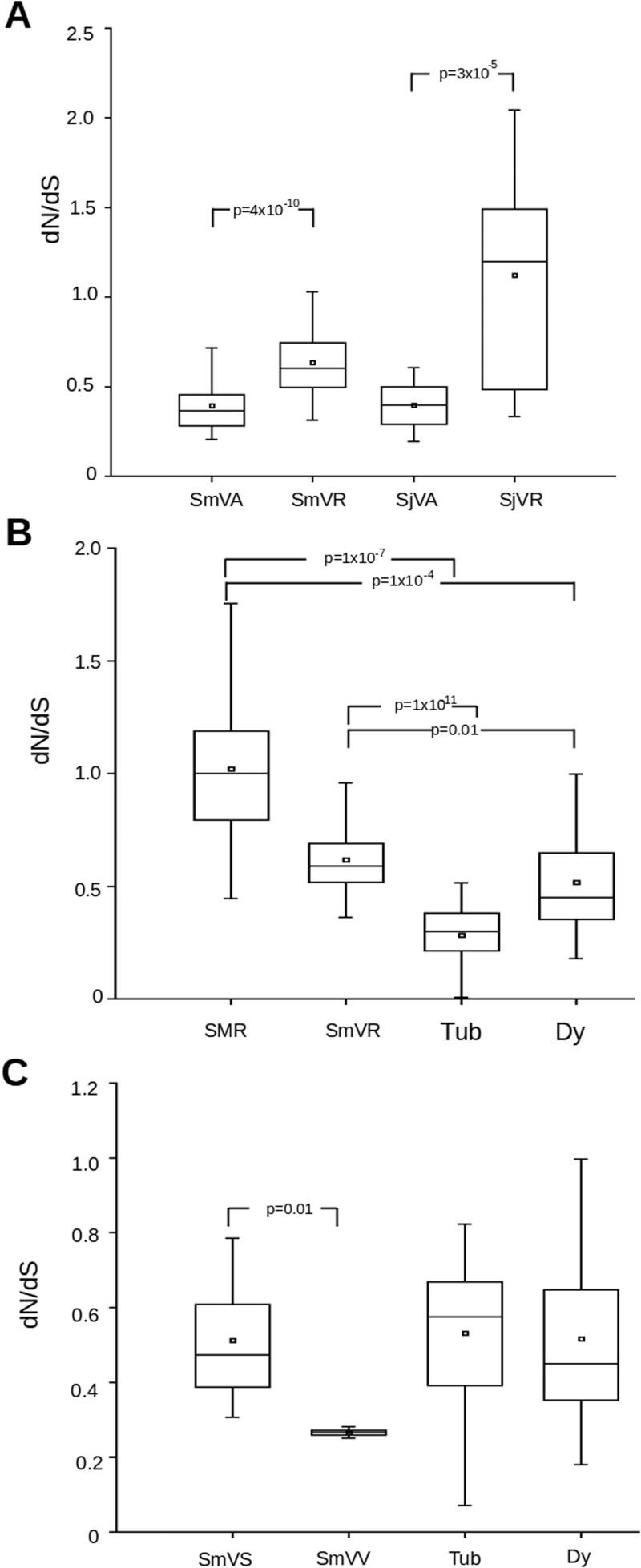
Distribution of d*N*/d*S* values for paralogous VAL genes. Box plots displaying d*N*/d*S* distributions relative to comparisons between: (*A*) Pairs of paralogous VAL genes classified as ancestrally divergent (occurred before the divergence of species) or as recently divergent (occurred after the divergence of species). SmVA, ancestrally divergent pairs in *S. mansoni* (*n* = 189); SmVR, recently divergent pairs in *S. mansoni* (*n* = 27); SjVA, ancestrally divergent pairs in *S. japonicum* (*n* = 64); SjVR, recently divergent pairs in *S. japonicum* (*n* = 16). (*B*) *Schistosoma* recently divergent paralogous MEG pairs (SMR; *n* = 12) and *S. mansoni* VAL genes pairs (SmVR; *n* = **27**) and from paralogous pairs from the recently expanding *S. mansoni* families Tubulin epsilon chain (Tub; *n* = 92) and Dynein light chain (Dy; *n* = 59). (*C*) Pair of *S. mansoni* paralogous VAL genes presenting high transcription levels at the intermediate snail host (SmVS; *n* = 4) or at the definitive vertebrate host (SmVV; *n* = 10) and from paralogous pairs from the recently expanding *S. mansoni* families Tubulin epsilon chain (Tub; *n* = 92) and Dynein light chain (Dy; *n* = 59). *P* values calculated using Wilcoxon signed-rank test for different comparisons between data sets are displayed.

Analysis of d*N*/d*S* from VAL paralog sequences was performed by separating pairs of sequences produced by a recent duplication from that formed by more ancestral divergences. A pair was considered recently duplicated when both sequences belonged to the same monophyletic group containing only sequences from the same organism in our phylogenetic analysis. It is notable that the d*N*/d*S* values of recently divergent pairs were significantly higher than ancestrally divergent ones in both *S. mansoni* and *S. japonicum* paralogs (fig. 5*A*). It is also noteworthy that values for recently divergent pairs of paralogs are higher than those observed for orthologs. Analysis of paralogous MEGs also indicates higher values for recently divergent pairs when compared with ancestral ones, but did not achieve statistical significance probably because of the limited number of cases compared (data not shown). It is also interesting that recently diverging pairs of schistosome MEGs and *S. mansoni* VALs display d*N*/d*S* values significantly higher than comparisons between members of two recently expanded families of genes in schistosomes (fig. 5*B*).

*VALs 2, 3, 5* and *9* from *S. mansoni* are mainly transcribed in the intermediate snail host, whereas *VALs 1, 4, 8, 10* and *12* have high transcript levels in stages associated with the invasion of or maturation in the definitive host (Chalmers et al. 2008). All these genes code for Group 1 VAL proteins that are likely to be secreted by the parasite but would be exposed to the intermediate and definitive host immune system, respectively. To detect the influence of the exposition to different immune system, measurements of d*N*/d*S* based on pairs of sequences within each of those groups of genes were performed (fig. 5*C*). Pairs of *S. mansoni* paralogs that code for VAL proteins likely to be exposed to the intermediate snail host display modest d*N*/d*S* values, whereas those exposed to the definitive host display significantly higher values. The values obtained for paralogs with evidence of exposure to the definitive host are comparable to those obtained for families of recently expanded genes in *S. mansoni*.

### Genes for Saposins Present in Parasite Vomitus Display High d*N*/d*S* Values

Analysis of d*N*/d*S* from genes corresponding to proteins previously detected in parasite vomitus using proteomic approach (Hall et al. 2011) allowed us to verify that 15 of 24 genes displayed values for the comparison between *S. mansoni* and *S. haematobium* genes equal to or below the average value of 0.28 seen for genes coding for predicted secreted proteins. Only three genes coding for saposins and one coding for a Niemann Pick C2 protein (NPC2) displayed d*N*/d*S* values higher than 0.5 in comparisons between *S. mansoni* and *S. haematobium* genes (table 2), comparable to those observed for MEGs and VALs. Curiously, a set of five genes coding for a Serpin, a cathepsin S, an alpha-macroglobulin, a saposin, and lysosome membrane-associated glycoprotein displayed relatively modest values (0.29–0.39) of d*N*/d*S* in comparisons between *S. mansoni* and *S. haematobium* genes, but relatively higher values (0.33–0.49) in comparisons between *S. mansoni* and *S. japonicum* genes.

**Table 2:** d*N*/d*S* Measurements for Genes Coding for Proteins Present at Adult Worm Vomitus

Description	Sma × Sha	Sma × Sja
Saposin Smp_130100	0.82	0.38
NPC-like cholesterol binding protein Smp_194840	0.76	0.35
Saposin Smp_014570	0.68	0.53
Saposin Smp_105450	0.56	0.53
Lysosome membrane-associated glycoprotein Smp_167770	0.39	0.49
Saposin Smp_194910	0.39	0.34
Alpha-2-macroglobulin Smp_089670	0.37	0.44
Cathepsin K/S Smp_139240	0.34	0.45
Serpin Smp_090080	0.29	0.40
Asparaginyl endopeptidase (Sm32) Smp_179170	0.28	0.27
Apoferritin Smp_063530	0.27	0.08
Dipeptylpeptidase I (Cathepsin C) Smp_019030	0.25	0.18
Lysosomal Pro-X carboxylpeptidase Smp_002600	0.22	0.14
Vesicle associated membrane protein Smp_136240	0.22	0.09
Cathepsin B1 isotype 1 (Sm31) Smp_103610	0.21	0.12
Dipeptylpeptidase II Smp_019030	0.20	0.08
Cathepsin B1 isotype 2 Smp_067060	0.20	0.13
Long-chain acyl-coenzyme thioesterase 1 Smp_150820	0.19	0.33
Ferritin-2 heavy chain, isoform 1	0.18	0.10
Glucan 1,4 beta-glucosidase	0.15	0.15
Ferritin-2 heavy chain, isoform 2 Smp_047650	0.15	0.11
DJ-1/PARK7-like protease Smp_082030	0.13	0.09
Calumenin, EF-hand Ca-binding protein Smp_147680	0.12	0.15
Ester hydrolase Smp_010620	0.10	0.10

Note.—Sma, *Schistosoma mansoni*; Sha, *Schistosoma haematobium*; Sja, *Schistosoma japonicum.*

### Two Surface-Exposed *S. mansoni* Vaccine Candidates Also Display High d*N*/d*S* Values

Considering that evolutionary pressure from the immune system was a possible factor promoting the increase in nonsynonymous substitution rates in VALs and MEGs, measurement of high values in current vaccine candidates would indicate that they had experienced similar pressure (table 3). Two vaccine candidates proposed for *S. mansoni*, Sm29 and the hydrophilic domain of TSP-2, shown to be at the parasite surface (Tran et al. 2006; Cardoso et al. 2008; Wilson 2012), display values that are comparable to those obtained for MEGs and VAL genes. In contrast, other vaccine candidates show d*N*/d*S* values near to or lower than the average for all genes (0.24 for *S. mansoni/S. haematobium*; 0.20 for *S. mansoni/S. japonicum*). It is also notable that d*N*/d*S* values for *Sm29* and *TSP-2* are higher in the comparisons between *S. mansoni* and *S. haematobium* genes than those observed for comparisons between *S. mansoni and S. japonicum* genes*.* No such trend is noticeable in when considering the assemblage of other vaccine candidates.

**Table 3:** d*N*/d*S* Measurements for Genes Coding for Vaccine Candidates

Vaccinal Candidate	Sma × Sha	Sma × Sja
Sm29	0.83	0.41
TSP-2	0.34 (0.65^a^)	0.12 (0.33^a^)
GST	0.32	0.16
SmTOR	0.30	0.20
Sjserpin	0.29	0.41
SjTGR	0.29	0.09
SjVLDL	0.27	0.11
Sm22.6	0.21	0.25
StoLP-2	0.20	0.05
Calpain	0.18	0.15
Sm21.7	0.18	0.53
SjCathepsin	0.15	0.13
GAPDH	0.14	0.06
SmRho	0.11	0.13
TPI	0.11	0.13
Sm14	0.11	0.06
Sm23	0.08	0.1
SOD	0.07	0.14
Aldolase	0.05	0.02
Sj22.7	0.04	0.004
14-3-3	0.03	0.19
Myosin heavy chain	0.02	0.03
Paramyosin	0.01	0.04

Note.—Sma, *Schistosoma mansoni*; Sha, *Schistosoma haematobium*; Sja, *Schistosoma japonicum.*

^a^Values corresponding to the analysis of the gene portion corresponding to the exposed hydrophilic loop utilized in vaccine trials.

**Fig. 6.—:**
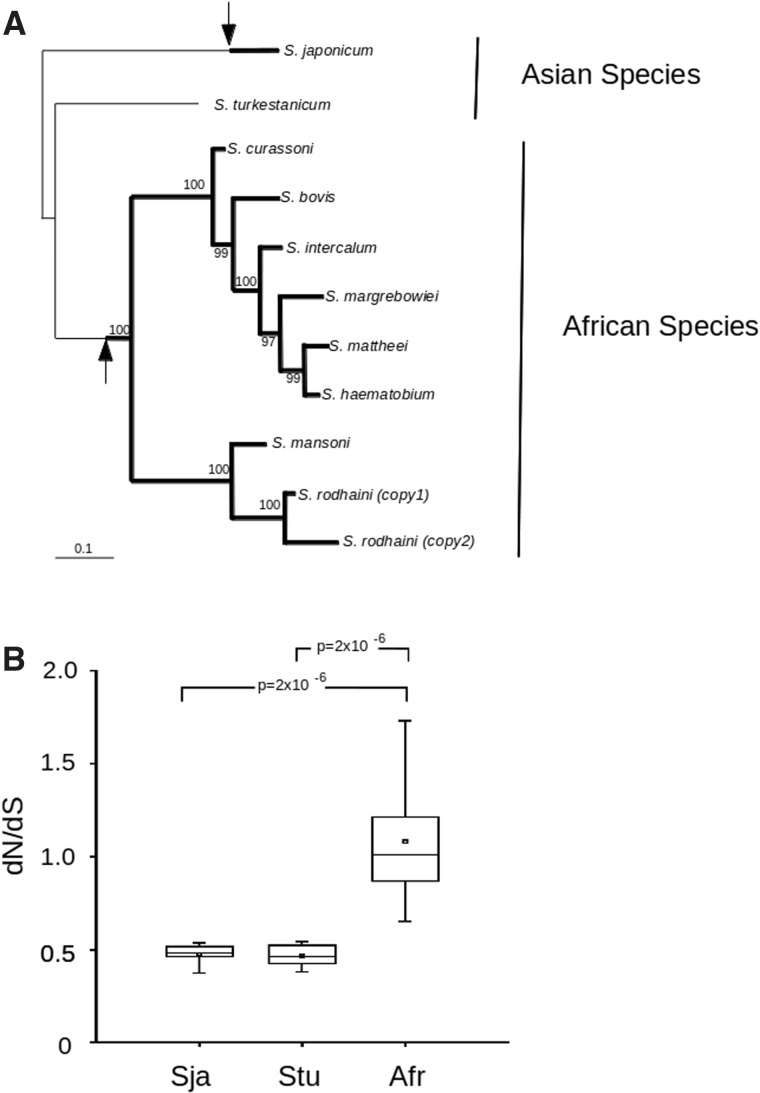
Evolution of Sm29 orthologs. (*A*) Phylogenetic analysis of Sm29 ortholog proteins from several Schistosome species using Bayesian inference. Numbers near nodes indicate calculated posterior probabilities. Thin and thick lines in the dendogram represent periods of low and high evolutionary pressure, respectively, in genes coding for proteins exposed to definitive host immune system. Arrows represent independent events that triggered the change from low to high evolutionary pressure regimen. Placement of the arrows reflect the fact that independent events must have occurred at the base of the branch representing African species and at some point of *S. japonicum* evolution, but are otherwise arbitrary. (*B*) Distribution of d*N*/d*S* values for populations corresponding to comparisons of *Sm29* orthologs: Sja, comparisons involving *S. japonicum Sm29* ortholog gene; Stu, comparisons involving *Schistosoma turkestanicum Sm29* ortholog gene; Afr, comparisons involving only African species. *P* values calculated using Wilcoxon signed-rank test for different comparisons between data sets are displayed.

To permit a better evaluation of the evolutionary pressure in these exposed proteins, we performed an analysis on the evolution of three conserved exons from *Sm29* ortholog genes of several species from the *Schistosoma* genus, recently made available at the Sanger ftp site. *Sm29* ortholog genes are single-copy in most of species studied (except for *Schistosoma rodhaini*, displaying two copies), which allows a more straightforward observation of the evolutionary pressure throughout the evolution of this genus. Phylogenetic analysis of *Sm29* sequences shows a monophyletic group of schistosomes of African origin (fig. 6*A*). Interestingly, values of d*N*/d*S* comparisons involving *Sm29* orthologs from Asian species were consistently lower than those involving solely orthologs from African species (fig. 6*B* and supplementary table S3, Supplementary Material online).

## Discussion

There is very clear evidence of recent gene duplication for several families of MEGs and for Group 1 VAL genes in all three schistosome species. Previous phylogenetic analysis of VAL genes reveals several species-specific monophyletic groups that display very short evolutionary distances (Cantacessi et al. 2012), being highly suggestive of recent duplication events. Considering that present versions of schistosome genomes are still fragmentary, improvement of the current assemblies might reveal novel members from these families.

It has previously been proposed that TE can be associated with segmental duplication either by promoting nonallelic homologous recombination (Zhang et al. 2013) or by generating double-strand breakages, thus providing homologous sites that will produce duplications in the repair process (Fiston-Lavier et al. 2007; Hedges and Deininger 2007). Therefore, description of higher frequencies of TE in the portions of *S. mansoni* genome harboring VAL genes and MEGs provides a possible mechanism for this notable recent expansion in their gene numbers.

The observed enrichment of TE could be a combination of two factors. The first is a preferential targeting of those elements to the regions harboring those genes. Second, gene duplications/rearrangements promoted by TE elements occur in genes encoding proteins apparently subjected to a high pressure from the environment. Thus, there is a greater chance of producing changes that will be positively selected and increase the odds of fixation of the insertions associated with those changes. It is notable that one event of exon duplication in the MEG-3 family was previously described as associated with the insertion of a TE (DeMarco et al. 2010), highlighting the potential for such events in shaping the structure of those genes. It has previously been proposed that TE plays a significant role in recent schistosome evolution due to the high transcriptional activity shown by some elements (DeMarco et al. 2004) and recent copy number bursts in some families (Venancio et al. 2010). Data presented here provide further support that these elements play an important role in the shaping of schistosome genome.

Interestingly, comparison between genes from VAL groups 1 and 2 in *S. mansoni* reveals a marked difference in introns sizes, those in Group 1 being much smaller. The presence of smaller introns would diminish the chance on an insertion of a TE within the gene structure. Within the context of a relatively unstable genomic region due to a higher than usual number of transposon copies, this would mean a significantly smaller chance that those genes would be subject of internal rearrangements. Indeed, Group 1 VALs tend to display a high number of genes with limited number of exons, whereas Group 2 tend to display a lower number of genes with more numerous exons.

It is worth noting that *VAL-6* (a Group 2 VAL) displays a portion of its gene with a structure very similar to that observed in MEGs (i.e., very small symmetrical exons) and it has been hypothesized that this gene in fact represents a composite structure formed by a recombination event between a VAL gene and an MEG (Verjovski-Almeida and DeMarco 2011). The fact that these two classes of gene display enrichment of similar TEs could certainly act as a facilitator of such an event.

In addition to the highly dynamic context observed in terms of generation of new copies, we also determined that MEGs and VAL genes displayed significantly higher d*N*/d*S* values when compared with other schistosome genes. This indicates that the genes are subject to an additional selective pressure not affecting regular genes. Several points strongly suggest that this additional pressure is caused by the host immune system: 1) Several protein products of MEGs and VAL genes have been described as produced by glands or epithelia exposed to the host immune system (Curwen et al. 2006; DeMarco et al. 2010; Li et al. 2013); 2) d*N*/d*S* values for the segment of the gene corresponding to mature MEG proteins are significantly higher than the portion corresponding to signal peptides. It should be noted that the portion of the gene corresponding to signal peptides in MEGs constitutes a good control for action of the evolutionary pressure of immune system, as signal peptides are destroyed within the endoplasmic reticulum; 3) d*N*/d*S* values for genes coding secreted VAL proteins (Group 1) are significantly higher than those of nonsecreted ones (Group 2) and also higher than VAL genes in *C. elegans*/*C. briggsae* comparisons. This indicates that high d*N*/d*S* values for Group 1 VAL genes cannot be attributed to any particular characteristics of their coded proteins, but instead should be related to their context; 4) Group 1 VAL genes coding for proteins exposed to the intermediate snail host display d*N*/d*S* values significantly lower than those exposed to the definitive vertebrate host and very similar to those observed for control genes, whereas VAL exposed to definitive vertebrate host have values similar to that observed for expanding *S. mansoni* families. This indicates that the snail immune system exerts practically no evolutionary pressure on these genes and that the evolutionary pressure postulated here must derive from specific characteristics of the definitive vertebrate host immune system.

In addition, two antigens (Sm29 and TSP-2) described as exposed at the tegument surface and lipid processing proteins present in the worm vomitus display values for d*N*/d*S* similar to those observed for MEGs and VAL genes. This provides further evidence of a general pressure from the immune system in exposed proteins rather than a phenomenon specific for MEG and VAL genes. It should be noted that the majority of the proteins previously described as at the surface of the schistosome tegument are proposed to be inaccessible to the host immune system due to the presence of a overlying membranocalyx (Braschi et al. 2006; Braschi and Wilson 2006). The low values of d*N*/d*S* observed for the majority of these proteins (which includes the vaccine candidate Sm23) would be a consequence of this relative inaccessibility. On the other hand, Sm29 has been proposed to be secreted to the exterior of the membranocalyx and SmTSP-2 has been proposed to be inserted in membranocalyx (Wilson 2012), which would render them exposed to the host immune system. The portion of *TSP-2* corresponding to the large extracellular loop shows much higher values than the rest of the gene, providing further evidence for a pressure of the immune system concentrated on exposed portions of a protein. The same trend has been observed for comparisons the extracellular loop region of *TSP-23* orthologs in schistosome species (Sealey et al. 2013), suggesting an analogous evolutionary process for *TSP-2 *and *TSP-23*. Moreover, polymorphism in the transcript sequences of *S. japonicum TSP-2* has recently been described and it has been suggested that at least a portion of this variation may be due to the presence of multiple copies (Zhang et al. 2011).

The proteins from parasite vomitus with high d*N*/d*S* values are saposins, lipid interacting proteins with cytolytic activity (Bruhn 2005), and NPC2, previously described as involved in binding of cholesterol (Xu et al. 2007). Microscopic analysis of erythrocyte ingestion in live parasites suggests that they are rapidly lysed in the parasite posterior esophagus (Li et al. 2013). Therefore it is possible that such lipid-interacting proteins would be part of the system responsible for destabilization of the erythrocyte membrane at very early stages of blood processing. That being the case, their location would make them relatively exposed to the host immune system, in contrast to other proteins present in a more internal location of the parasite digestive system.

It should be noted that if indeed high rates of nonsynonymous changes are caused by pressure from the immune system, this would imply that during evolution, definitive hosts were once able to mount effective responses involving these antigens. The fact that two vaccine candidates proposed for *S. mansoni* display such high rates gives hope that it would be possible to artificially stimulate an analogous protective response based on these antigens. On the other hand, because such antigens are subjected to a great evolutionary pressure, a high level of sequence heterogeneity is expected within natural populations, which may constitute a significant obstacle to development of effective vaccines. Several of the previously proposed vaccine candidates display very low rates of nonsynonymous changes, which may be related to the fact that they are not predicted to be exposed at the schistosome surface, but are located internally. These low d*N*/d*S* rates would imply that a naturally protective response could not be mounted against such cytosolic antigens. This does not necessarily mean that these cytosolic antigens could not induce high protection levels by artificial means, but certainly raises questions about which mechanisms would account for this supposed differential response. Taken together, this suggests that evaluation of d*N*/d*S* rates and number of gene copies in the genomes could be interesting new parameters to evaluate novel vaccine candidates.

**Fig. 7.—:**
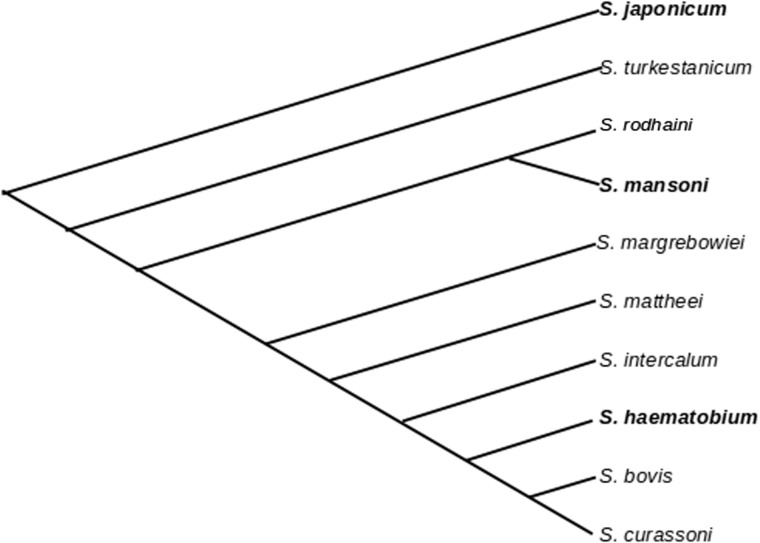
Cladogram showing the relationship of *Schistosoma* species studied in this work, adapted from the analysis of mitochondrial markers from *Schistosoma* genus presented in Lawton et al. (2011). The three species extensively studied in this work are in bold.

Analysis of d*N*/d*S* of *Sm29* orthologs from several species of schistosomes indicates that comparisons involving only African species tend to generate higher values than those in which at least one of the genes is from an Asian species; This is concordant with the scenario observed in MEGs and VALs in the three studied species. Moreover, the values of d*N*/d*S* obtained for the comparison of *Sm29* orthologs from the two Asian species (0.34) are lower than any of the other comparisons made (supplementary table S3, Supplementary Material online), and relatively near to the average value found for comparisons involving regular genes (∼0.22). This would in principle suggest that genes coding for exposed proteins from Asian species are subject to much less evolutionary pressure from the host immune system. It is also noteworthy that in the analysis of MEGs and VAL genes d*N*/d*S* values for *S. mansoni* × *S. haematobium* comparisons are consistently higher than *S. mansoni* × *S. japonicum* comparisons, which is again consistent with a lower evolutionary pressure in Asian species. However, such a view is not in agreement with the data showing that recently divergent *S. japonicum* paralogs display high d*N*/d*S* values, indicating a high evolutionary pressure. These two observations can be reconciled if we assume that *S. japonicum* has only recently been subjected to an increased evolutionary pressure. As *S. japonicum* was the first species to diverge, a long period of time was spent under a low evolutionary pressure regime (fig. 7), thus explaining the lower apparent rate of nonsynonymous changes in ortholog comparisons involving this species. Additionally, such a model would explain the higher d*N*/d*S* values observed for recently divergent paralog pairs when compared with ancestrally divergent ones.

Considering we verified in VALs from Group 2 and MEGs that both the expansion in number of copies and the increase in the rate of nonsynonymous substitutions are relatively recent, it is not unreasonable to suppose that evolutionary pressures leading to these two trends were triggered by the same cause. The fact that at least three different classes of genes (VALs, MEGs, and TSP-2) follow the same pattern of recent increase of copy numbers associated with higher nonsynonymous substitutions rates increases the likelihood that this might represent a single coordinated response to an evolutionary pressure.

The change that led to the transition from a low evolutionary pressure regimen to a high one must have occurred in an independent manner in an ancestor of the African species and in an ancestor of *S. japonicum*. As most of the duplication events in *S. mansoni* and *S. haematobium* are species-specific, it is possible to speculate that the change to a high evolutionary pressure regimen in African species occurred a short time before the divergence of the two evolutionary branches containing each of these species (fig. 6*A*). It has been hypothesized that the African branch of species studied here was directly derived from ancestors that were similar to *S. turkestanicum* (Lawton et al. 2011). The fact that this species displays evidence of lower evolutionary pressure in the analysis of *Sm29* orthologs suggests that the factors that triggered the change to a high evolutionarily regimen might have occurred during the migration of *Schistosoma* species to Africa. If we consider that the immune system of the definitive host is responsible for the increased evolutionary pressure, it is reasonable to assume that the existence of periods of low and high evolutionary pressure might be correlated with the association of the parasite with definitive hosts that display different levels of ability to mount an effective immune response against those exposed proteins. The fact that a change in evolutionary pressure possibly occurred during establishment of *Schistosoma* species in Africa might reflect the natural process of adaptation of the parasite in a new environment where new types of hosts are available.

The data presented here are a first step to better understand the process of coevolution of schistosome-exposed proteins and the definitive host immune system. The differential pressure in the course of evolution permits the hypothesis to be proposed that some definitive hosts are able to mount a more effective response based on those exposed antigens. Further studies on differential immune responses against such antigens in different classes of definitive host may help to clarify a possible immunological mechanism that is associated with this process and may be very important in the formulation of vaccine strategies that enable the human immune system to fight this parasite. The fact that several external antigens are exposed to a diversifying selection could imply that several polymorphic forms might be distributed within parasite populations, arguing for more detailed studies on variation of these antigens in natural populations. This might indicate that there is a definitive tendency of truly exposed antigens to diversification, creating a deadlock in which vaccines formulated by exposed antigens would not be effective due to polymorphisms in the natural population, whereas nonvarying antigens would actually represent nonexposed antigens for which no effective immune response could be mounted. Further inquires in this subject seem imperative to allow a more rational approach to vaccine development in schistosomes. That being the case, this could represent a hurdle to vaccine development based on these antigens, as vaccines based on a single polymorphic form might not be effective against a heterogeneous population.

## Supplementary Material

Supplementary file S1, figure S1, and tables S1–S3 are available at *Genome Biology and Evolution* online (http://www.gbe.oxfordjournals.org/).

Supplementary Data
