# Supplementary material for: Accelerated Evolution of Schistosome Genes Coding for Proteins Located at the Host–Parasite Interface
Source: Genome Biol Evol. 2015 Jan 6;7(2):431–43. doi: 10.1093/gbe/evu287 (PMC4350168; doi:10.1093/gbe/evu287)
Supplement: Supplementary Data [file supp_7_2_431__index.html]

Accelerated evolution of Schistosome genes coding for proteins located at the host-parasite interface. — Accelerated Evolution of Schistosome Genes Coding for Proteins Located at the Host–Parasite Interface — Supplementary Data 

# Accelerated Evolution of Schistosome Genes Coding for Proteins Located at the Host–Parasite Interface

## Supplementary Data

files

**Files in this Data Supplement:**

- Supplementary Data - xls file
- Supplementary Data - doc file
- Supplementary Data - xls file
