# Supplementary material for: Accelerated Evolution of Schistosome Genes Coding for Proteins Located at the Host–Parasite Interface
Source: Genome Biol Evol. 2015 Jan 6;7(2):431–43. doi: 10.1093/gbe/evu287 (PMC4350168; doi:10.1093/gbe/evu287)

**
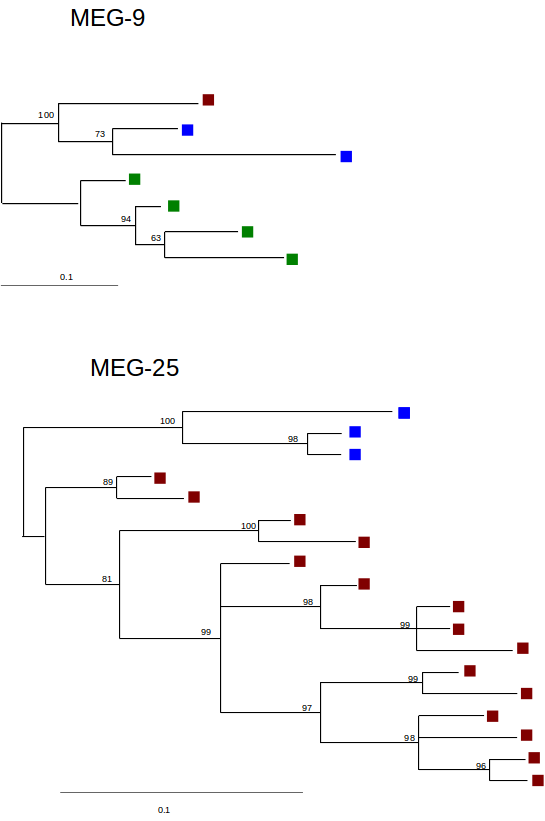
**

**Supplementary Figure 1-** Phylogenetic analysis of MEG family members from schistosomes using Bayesian inference. Analysis was based on the alignment of a long conserved exon sequence from those genes. Squares represent genes from *S. manson*i (red), *S. haematobium* (blue) and *S. japonicum* (green). Numbers near nodes indicate calculated Bayesian posterior probabilities.

**Supplementary table 2**- Frequency of transposable elements within the vicinity of MEG and VAL genes.

| Transposable element | Gene set | Verified copies | Expected copies | Z-value^1^ |
| --- | --- | --- | --- | --- |
| Sm | MEGs | 140 | 82 | 6.41 |
| Sm | VALs | 91 | 52 | 5.41 |
| Sm-alpha | MEGs | 88 | 53 | 4.81 |
| Sm-alpha | VALs | 44 | 33 | 1.92 |
| Perere-3 | MEGs | 57 | 38 | 3.08 |

1|Z| > 3, 0902 : p < 0, 001; |Z| > 2, 3263 : p < 0, 01; |Z| > 1, 6449 : p < 0, 05.


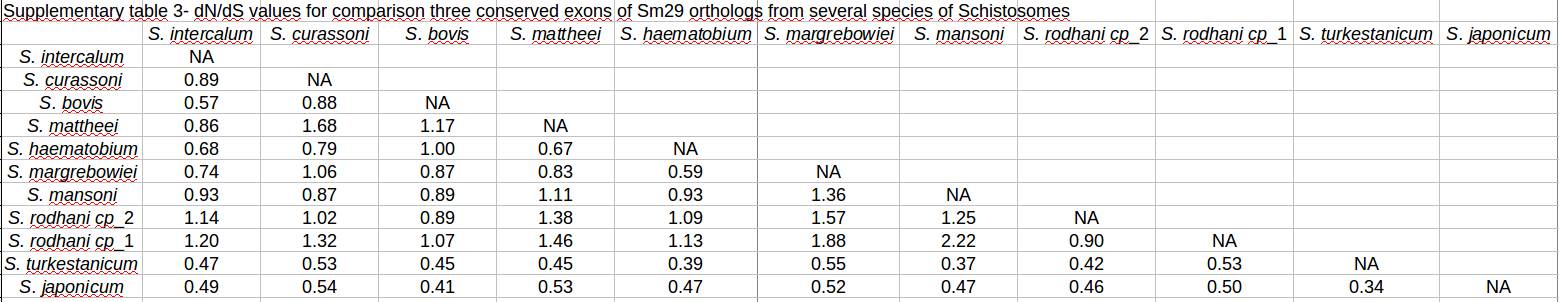

Supplement: Supplementary Data [file supp_evu287_supplementary_material.doc]
